# *Polygonatum cyrtonema* Polysaccharides Alleviates Diet-Induced Obesity in Mice by Modulating the Gut Microbiota and Reducing Intestinal Inflammation

**DOI:** 10.4014/jmb.2512.12028

**Published:** 2026-05-15

**Authors:** Rongting Yang, Meiqi Dong, Jingbo Wu, Lijuan Wang, Yapeng Liu, Mengyu Cong, Juan Liang

**Affiliations:** 1 School of Pharmacy, Anhui University of Chinese Medicine, Hefei 230012, P. R. China; 2 Heilongjiang International Travel Health Care Center, Harbin, Heilongjiang 151000, P. R. China

**Keywords:** *Polygonatum cyrtonema* polysaccharide, Gut microbiota, Short-chain fatty acids, intestinal inflammation, intestinal barrier function

## Abstract

This study examined the potential function and mechanism of *Polygonatum cyrtonema* polysaccharide (PCP) in alleviating high-fat diet -induced obesity in mice. Our results showed that high-dose PCP (PCP-H) significantly reduced body weight, blood lipid, and blood glucose levels in HFD-induced obese mice, whereas low-dose PCP (PCP-L) exhibited limited efficacy. Gut microbiota analysis revealed that PCP-H interventions significantly increased the relative abundance of beneficial bacteria, including *Muribaculaceae*, *Bacteroides*, and *Alloprevotella*, and decreased the relative abundance of pathogenic bacteria, such as *Mucispirillum* and *Escherichia*-*Shigella*, in HFD-fed obese mice. Additionally, PCP-H administration increased the levels of short-chain fatty acids (SCFAs) such as acetic acid, propionic acid and butyric acid, which were correlated with the regulatory effect of PCP-H on the microbiota composition. Furthermore, PCP-H downregulated the mRNA expression of TLR-4, TNF-α, IL-1β, and IL-6 in the intestines, resulting in attenuated intestinal inflammation. Moreover, PCP-H restored intestinal barrier function by upregulating the gene and protein expression levels of claudin-1, occludin and ZO-1, which in turn led to decreased serum levels of proinflammatory cytokines (*e.g.*, TNF-α, IL-1β, and IL-6). Taken together, these findings indicate the effect of PCP-H on mitigating diet-induced obesity may be associated with elevated levels of SCFAs, decreased intestinal inflammation, and enhanced intestinal barrier function, potentially mediated through the regulation of gut microbiota.

## Introduction

The rapid advancement of society and economy, along with significant shifts in dietary patterns, have led to an increasing prevalence of overweight and obesity [1].There are an estimated over 1 billion individuals suffering from overweight and obesity worldwide, which is projected to surge by around 50% by 2035 [2]. Current evidence indicates that obesity is associated with dysregulated glucose and lipid metabolism, resulting in various metabolic disorders [3]. Consequently, obesity has become a major global public health concern. Western medicines are predominantly used for the treatment of obesity. However, most Western weight loss medications can only be administered for a limited time and frequently come with side effects. Additionally, weight rebound is prone to occur following drug discontinuation [4-6]. It is therefore vital to identify a safe and effective traditional Chinese medicine (TCM)-derived substance for obesity intervention and elucidate its mechanism of action for improving the clinical management of obesity.

*Polygonatum cyrtonema* is a perennial herb of the Liliaceae family that has been used in China as a medicinal and dietary substance for over 2,000 years. Clinical and pharmacological studies have confirmed the efficacy of *P. cyrtonema* in promoting weight loss and decreasing lipid levels [7, 8]. Polysaccharides are the primary active ingredients of *P. cyrtonema*. A study by Gu *et al*. demonstrated that oral gavage of *P. kingianum* polysaccharides significantly inhibited weight gain, alleviated diabetic symptoms, and reduced blood lipid levels in high-fat diet (HFD)-induced obese rats [9]. Our previous work also revealed that *P. cyrtonema* polysaccharide (PCP) intervention reduced weight and weight gain by 15.4% and 30.4% in HFD-fed obese mice, respectively. In addition, PCP treatment significantly decreased adipocyte size, inflammatory cell infiltration, and accumulation of fat droplets in the liver, demonstrating that PCP intervention effectively ameliorated HFD-induced obesity [10]. However, the mechanism by which PCP regulates obesity remains unclear and requires further investigation.

The human gut hosts approximately trillions of microorganisms collectively known as the gut microbiota. There is growing evidence indicating that the gut microbiota plays a critical role in obesity and associated metabolic diseases by participating in nutrient absorption and energy regulation processes[11, 12]. Turnbaugh *et al*. found significant differences in microbiota composition between obese individuals and healthy controls subjected to identical conditions (All twins were 25–32 years old, of European or African ancestry, were generally concordant for obesity or leanness, and had not taken antibiotics for at least 5.49 ± 0.09 months.) [13]. Other researches showed that adult germ-free mice did not develop an obese phenotype when subjected to a high-fat diet. However, when the intestinal flora of obese mice was transplanted into germ-free mice and continued to be fed HFD, the development of obesity was observed [14–16]. These results suggest a strong correlation between obesity and the composition of gut microbiota.

Short-chain fatty acids (SCFAs) are key metabolites produced by the gut microbiota that play a beneficial role in the regulation of host energy metabolism, especially in obesity and related metabolic diseases [17]. Lan *et al*. reported that seabuckthorn polysaccharide mitigates HFD-induced obesity via the microbiota-SCFAs-liver axis [18]. Intestinal epithelial cells (IECs) form the intestinal barrier through a network of tight junction proteins such as occludin, claudin, and ZO [19]. Sang *et al*. demonstrated that polysaccharides extracted from the sporoderm-broken spore of *Ganoderma lucidum* prevents HFD-induced obesity in mice by restoring gut dysbiosis and intestinal barrier functions [20]. Similarly, crude isinglass polysaccharides can attenuate HFD-induced obesity in mice by decreasing serum levels of proinflammatory cytokines, increasing the abundance and diversity of the gut microbiota, and regulating the expression of proteins associated with the intestinal barrier [21]. Taken together, these findings suggest that polysaccharides can mitigate HFD-induced obesity by regulating gut microbiota and metabolites, and can reduce intestinal inflammation, thereby improving intestinal barrier function.

However, there is still a lack of in-depth study on how PCP regulates intestinal flora to improve obesity. Therefore, this study aims to investigate the weight loss and fat reduction mechanisms of PCP based on intestinal flora using diet-induced obesity in mice by a high-fat diet as an experimental model.

## Materials and Methods

### Materials and Reagents

The *Polygonatum cyrtonema* Hua rhizomes (lot number: 21319) were purchased from Anhui Qingyang Jiuhua Traditional Chinese Medicine Technology Co., Ltd (China). High-fat diet was purchased from Changzhou Rat One Rat Two Biotechnology Co., Ltd. (China). Triglyceride (TG), total cholesterol (TC), high-density lipoprotein cholesterol (HDL-C), and low-density lipoprotein cholesterol (LDL-C) test kits were purchased from Nanjing Jiancheng Bioengineering Institute (China). TNF-α, IL-6 and IL-1β ELISA kits were provided from Multi Sciences Biotech Company (China). β-actin, occludin, ZO-1 and claudin-1 antibodies were purchased from Beyotime Biotechnology Co., Ltd. (China). Analytical grade reagents were used in all experiments. Qubit dsDNA Assay Kit (Lot No.: Q32854) was purchased from Life Technologies (USA). MagPure Stool DNA LQ Kit (Lot No.: D6356-02) was purchased from Magen Biotechnology Co., Ltd. (China). Tks Gflex DNA Polymerase (Lot No.: R060B) was purchased from Takara Bio Inc. (China).

### Preparation of PCP

PCP was extracted following the protocol reported in our previous study[22]. Briefly, dried *P. cyrtonema* was ground into powder, mixed with water at a 1:10 ratio (w/v), heated and extracted twice by reflux extraction for 2 h, concentrated to 1/10 of its original volume, and precipitated with 95% ethanol. After centrifugation (4000 rpm for 10 min), the supernatant was removed, and the precipitate was collected and lyophilized to obtain PCP, with a extraction yield of 25.7%. The structure characteristics of PCP were analyzed and reported in our previous publication[22] as the PCP used in the present study was prepared via the same extraction and purification protocol.

### Animals and Experimental Design

**Fig. 1:**
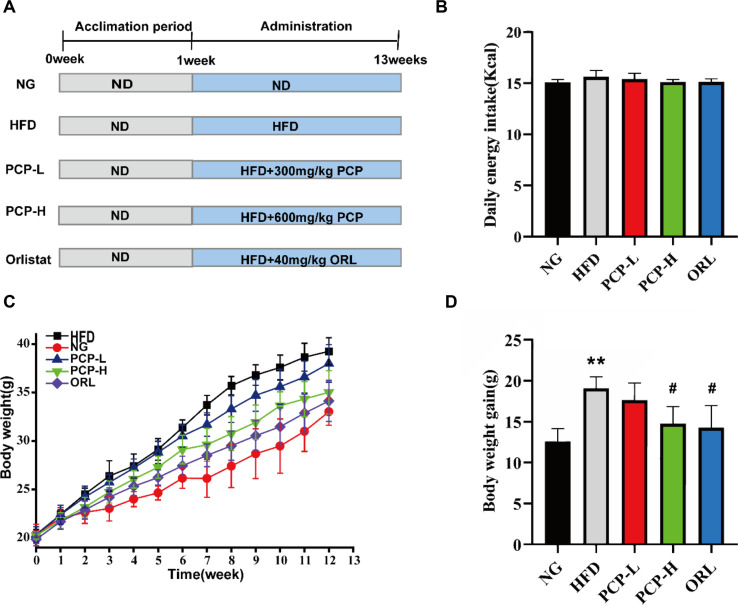
Effect of PCP intervention on HFD-diet mice. After a 1-week acclimation period, mice were randomly assigned to experimental groups. NG (control group), HFD (high-fat diet group), PCP-L (low-dose PCP group), PCP-H (high-dose PCP group), ORL (orlistat group). (**A**) Experimental design. (**B**) Average energy intake. (**C**) Growth curve of body weight (**D**) Weight gain. Data were shown as mean ± SD (n = 8). *p < 0.05, **p < 0.01 compared with Control group, and ^#^*p* < 0.05, ^##^*p* < 0.01 compared with HFD group.

SPF C57BL/6 male mice (8 weeks old, n = 50) were purchased from Hangzhou Ziyuan Experimental Animal Technology Co., Ltd. (China). The animals were housed in a barrier facility at 24 ± 2°C with 55% humidity and 12 h/12 h light/dark cycle. All animal procedures were performed according to the national legislation and the Laboratory Animal Ethics Committee of Anhui University of Chinese Medicine (AHUCM-mouse-2021103). After a 1-week acclimation period, mice were randomly divided into the control group (NG), HFD group, low-dose PCP (PCP-L) group, high-dose PCP (PCP-H) group, and orlistat (ORL) group. Mice in the control group were fed a standard diet, whereas those in the other four experimental groups were fed HFD. The composition of the high-fat diet is shown in Table 1, while that of the normal chow is provieded in Supplementary Table 3. For treatment, the control and HFD groups were given 5% CMC-Na via oral gavage; the PCP-L and PCP-H groups were orally administered 300 mg/kg and 600 mg/kg body weight PCP, respectively?and the doses selected based on the Chinese Pharmacopoeia (2020 edition) and our previous research [22, 23]; and the ORL group was given 40 mg/kg of ORL (Fig. 1A). All treatments were administered for 12 weeks. The daily dietary intake and weekly body weight of mice were monitored. After 12 weeks, all mice were fasted for 12 h, anesthetized with 1% sodium pentobarbital (25 mg/kg), and then used for sample collection. Colon contents and serum were collected and stored at -80°C, and ileal tissue was fixed in 4% paraformaldehyde for subsequent analyses.

### Biochemical Analysis

The serum levels of TG, TC, HDL-C, LDL-C, TNF-α, IL-6, and IL-1β were quantified according to the respective kit protocols. Fasting blood glucose (FBG) levels were assessed using a blood glucose meter (Roche Diagnostics, Germany).

### Histopathology

Ileal tissues were fixed in 4% paraformaldehyde for 24 h, dehydrated through a graded ethanol series (80%, 85%, 90%, 95%, and 100%), cleared in xylene, and embedded in paraffin. Sections were cut at 4 μm thickness, deparaffinized, and stained with hematoxylin and eosin (H&E). Histological examination was performed using a light microscope (Olympus-IX73-DP80, Japan). For each sample, villus height and crypt depth were measured in at least five well-oriented villus-crypt units using ImageJ software, and the villus height/crypt depth ratio was calculated to assess intestinal morphology.

### Microbiota Analysis by 16S rRNA Sequencing

Total DNA was extracted from colonic contents using the MagPure Stool DNA LQ Kit. DNA concentration and purity were assessed with a NanoDrop 2000 and agarose gel electrophoresis, and normalized to 5 ng/μL. The V3-V4 region of the 16S rRNA gene was amplified using primers 343F (5'-TACGGRAGGCAGCAG-3') and 798R (5'-AGGGTATCTAATCCT-3'). After two rounds of PCR, products were purified with AMPure XP beads, quantified by Qubit, and sequenced on an Illumina NovaSeq 6000 platform (OE Biotech, China).

Primers were removed from the raw sequences using Cutadapt. The QIIME 2 platform was used to call DADA2 for quality filtering, denoising, assembly, and chimera removal, yielding representative sequences and the ASV abundance table. Genus-level annotation was completed using q2-feature-classifier and the Silva 138 database. QIIME 2 software was used to analyze species richness, α-diversity, and β-diversity, and conduct inter-group statistical comparisons; to further identify the biomarker species in each experimental group, the software was continuously used for LEfSe (i.e., linear discriminant analysis (LDA) coupled with effect size measurements) analysis. The key functional flora were screened by comparing the flora differences among the NG group, HFD group, and PCP-H group. Kruskal-Wallis test and Wilcoxon test were used to determine the biomarker species with significant differences between groups, and linear discriminant analysis (with LDA score > 3) was employed to evaluate the score influence of each significantly different biomarker species.

### Analysis of SCFA Levels

Approximately 50 mg of fecal sample was mixed with 500 μL of ethyl ether, homogenized in an ice bath for 1 min, and centrifuged to obtain the supernatant. The supernatant was then mixed with sodium phosphate (15%), 4-methylvaleric acid (375 g/mL) and ethyl ether (280 μL), homogenized in an ice bath for 1 min, and centrifuged again to obtain the supernatant for determination. The concentrations of SCFAs were determined using a Thermo Trace 1300 Gas Chromatograph (GC) System (Thermo Fisher Scientific, USA) equipped with a HP-INNOWAX column (30 m × 0.25 mm × 0.25 μm). The GC conditions were 1 μL injection volume, 250°C inlet temperature, and 10:1 distribution ratio. The temperature program was set at 90°C, ramp 10°C /min to 120°C, ramp 5°C /min to 150°C, and ramp 25°C /min to 250°C for 2 min. The mass spectrometry (MS) parameters were 300°C ion source temperature, 250°C transfer line temperature, 70 eV electron energy, and selected ion monitoring (SIM). Standard substances used were acetic acid, propionic acid, isobutyric acid, butyric acid, isovaleric acid, and caproic acid. Quantitative analysis was performed by comparing the peak areas of the samples with that of the internal standard.

### Detection of Intestinal mRNA Expression Level by RT-PCR

RNA was isolated from the intestinal tissue using Trizol, sequentially precipitated by chloroform and isopropanol, and reverse transcribed into cDNA using the PrimeScript™RT Kit with gDNA Eraser. The mRNA expression of TLR-4, TNF-α, IL-1β, IL-6, claudin-1, occludin, and ZO-1were quantified by RT-PCR with the StepOne Plus Real-Time PCR System. β-actin was used as the reference gene. Relative gene expression was calculated using the 2^-ΔΔCT^ method. Details of the RT-PCR primer sequences are shown in Supplementary Table S1 and S2.

### Western Blot

Intestinal tissues were lysed with 100 μL of RIPA Lysis Buffer mixture (cell lysate/protease inhibitor/phosphatase inhibitor ratio of 100:10:1), and proteins were extracted and quantified by a BCA kit. Equal amounts of protein samples were separated using SDS-PAGE, transferred onto a PVDF membrane, incubated with ZO-1, occludin, claudin-1, and β-actin primary antibodies overnight at 4°C, and washed 3 times with 1% TBST. After washing extensively, the membrane was incubated with secondary antibodies conjugated to horseradish peroxidase (HRP) at room temperature for 2 h. Finally, protein bands were quantified by Image J and normalized to β-actin.

### Statistical Analysis

Statistical analysis was performed using SPSS 26.0 and GraphPad Prism 8.3 software. Data were expressed as the mean ± standard deviation (SD). One-way analysis of variance (ANOVA) followed by Tukey’s test was used to evaluate differences among groups. Different lowercase letters indicate significant differences (*p* < 0.05), while the same letter indicates no significant difference (*p* > 0.05). A *p*-value < 0.05 was considered statistically significant. In addition, *p < 0.05, **p < 0.01, ***p < 0.001, and ****p < 0.0001 represent statistically significant differences compared with the Control group; ^#^*p* < 0.05, ^##^*p* < 0.01, ^###^*p* < 0.001, and ^####^*p* < 0.0001 represent statistically significant differences compared with the HFD group.

## Results

### PCP-H Reduced Weight of HFD-Induced Obese Mice

As shown in Fig. 1C, the body weight of mice was similar among the five groups at baseline and became significantly higher in the HFD group (18.9% increase) than in the control group at 12 weeks (*p* < 0.01), indicating successful induction of obesity. Compared to the HFD group, both the PCP-H and ORL groups, but not the PCP-L group, showed significantly lower body weight (*p* < 0.05). The weight gain of mice in each group at the end of the 12-week (Fig. 1C) exhibited a comparable trend to that observed in Fig. 1D, which demonstrates that high-dose PCP and ORL effectively mitigated HFD-induced obesity in mice. There was no significant difference in daily energy intake among the groups (Fig. 1B), which indicated that the weight loss effect of PCP-H was independent of energy intake.

### PCP-H Regulates Blood Lipids and Blood Glucose in Obese Mice

**Fig. 2:**
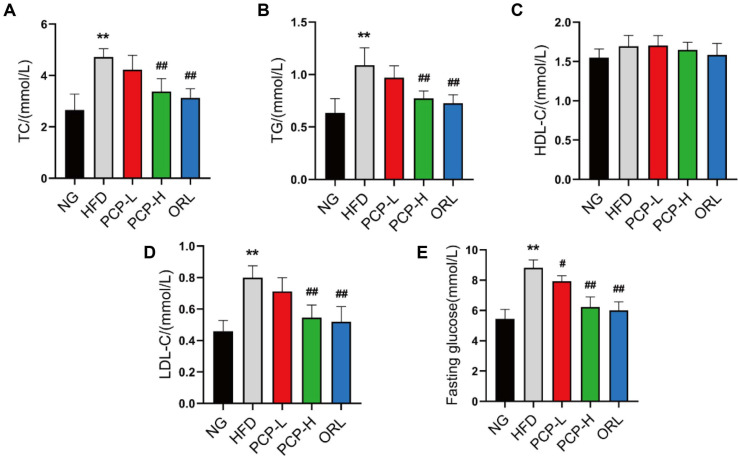
Effect of PCP on blood lipid and blood glucose. NG (control group), HFD (high-fat diet group), PCP-L (low-dose PCP group), PCP-H (high-dose PCP group), ORL (orlistat group). (**A**) TC (total cholesterol), (**B**) TG (Triglyceride), (**C**) HDL-C (high-density lipoprotein cholesterol), (**D**) LDL-C (low-density lipoprotein cholesterol), (**E**) Fasting glucose. Data were shown as mean ± SD (n = 8). *p < 0.05, **p < 0.01 compared with Control group, and ^#^*p* < 0.05, ^##^*p* < 0.01 compared with HFD group.

Analyses of blood lipids and blood glucose revealed significantly higher levels of TC, TG, LDL-C and FBG in the HFD group compared with the control group (*p* < 0.01) (Fig. 2A, 2B, 2D and 2E). Low-dose PCP intervention significantly reduced FBG levels in obese mice (*p* < 0.05), but had no effect on TC, TG and LDL-C levels. Conversely, both high-dose PCP and ORL interventions led to significantly decreased TC, TG, LDL-C and FBG levels in obese mice (*p* < 0.01). Collectively, these data suggest that PCP-H intervention effectively ameliorates glucose and lipid metabolism disorders in HFD-fed obese mice, whereas PCP-L exhibited limited efficacy.

### PCP-H Modulates the Gut Microbiota and SCFA Levels

**Fig. 3:**
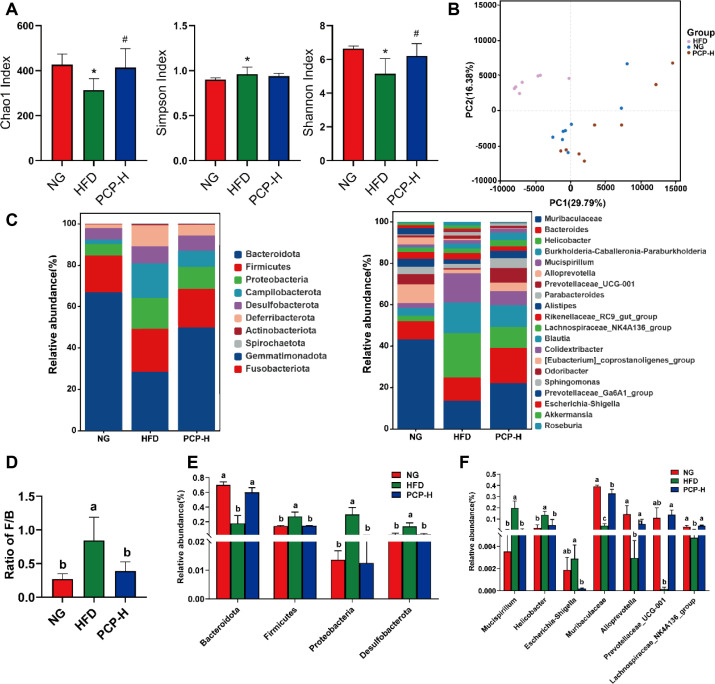
Effect of PCP on alpha and beta diversity. NG (control group), HFD (high-fat diet group), PCP-H (high-dose PCP group). (**A**) Chao1index, Simpson index, Shannon index, (**B**) PCoA diagram of beta diversity. (**C**) Relative abundance of intestinal microbiota at phylum level and genus level. (**D**) Ratio of *Firmicutes* to *Bacteroidetes* in the gut microbiota. (**E**) The relative abundance and ratio of enriched phyla. (**F**) The relative abundance and ratio of enriched genera. Data were shown as mean ± SD (n = 8). *p < 0.05 compared with Control group, and ^#^*p* < 0.05 compared with HFD group.

Since the PCP-L group (300 mg/kg) had a limited effect on improving the body weight and blood lipid indices (TC, TG, LDL-C) of experimental animals in the preliminary pharmacodynamic evaluation, this group was not included in the subsequent study. In addition, the mechanism of action of the ORL group is to reduce body weight by inhibiting intestinal lipase activity and decreasing total cholesterol and triglyceride levels [24, 25], which is significantly different from the core research direction of this study that “PCP exerts anti-obesity effects by regulating the microbiota-intestinal barrier axis”. Therefore, it was not included in the subsequent mechanistic analysis. Based on this, this study selected the control group (NG), obesity model control (HFD), and high-dose PCP group (PCP-H) to evaluate the effect of PCP on the intestinal flora. Analysis of alpha diversity using the Chao1, Simpson, and Shannon indices showed that the HFD group had decreased microbial richness and diversity, which was significantly reversed by PCP-H intervention (Fig. 3A). Principal coordinate analysis demonstrated that the microbiota composition was distinct between the HFD group and the other groups. Furthermore, the microbiota composition of the PCP-H group overlapped with that of the control group, indicating that high doses of PCP-H may restore the gut microbiota in obese mice (Fig. 3B).

Intestinal flora structure analysis showed *Bacteroidota*, *Firmicutes*, *Proteobacteria*, *Campylobacterota*, *Desulfobacterota* and *Deferribacterota* were the predominant phyla in mice (Fig. 3C). HFD induced a significant decrease in the relative abundance of *Bacteroidota* (*p* < 0.01) and an increase in the relative abundance of *Proteobacteria* and *Desulfobacterota* (*p* < 0.05), along with a markedly increased *Firmicutes*/*Bacteroidetes* ratio (Fig. 3D). In contrast, PCP-H intervention effectively prevented these alterations and restored the microbiota to a composition similar to that of the control group. Significant differences in abundance at the genus level were also observed among the groups (Fig. 3C and 3F). *Muribaculaceae*, *Bacteroides*, *Helicobacter*, *Burkholderia*-*Caballeronia*-*Paraburkholderia*, *Mucispirillum*, *Alloprevotella*, *Prevotellaceae_UCG-001*, *Parabacteroides*, *Alistipes*, *Rikenellaceae_RC9*_gut_group, *Lachnospiraceae_NK4A136*_group, *[Eubacterium]*_coprostanoligenes_group and *Prevotellaceae_ Ga6A1*_group were the predominant genera (> 1% abundance) in mice. Compared with the control group, the HFD group had significantly decreased *Muribaculaceae*, *Alloprevotella* and *Lachnospiraceae_NK4A136*_group (*p* < 0.05) as well as increased *Mucispirillum* and *Helicobacter* (*p* < 0.01). In addition, HFD resulted in increased *Escherichia*-*Shigella* and decreased *Prevotellaceae_UCG-001* compared with normal diet, but these changes were not statistically significant (Fig. 3F). On the other hand, PCP-H intervention resulted in varying levels of restoration in the relative abundance of the above genera, leading to a microbial community composition that more closely resembled that of the control group.

**Fig. 4:**
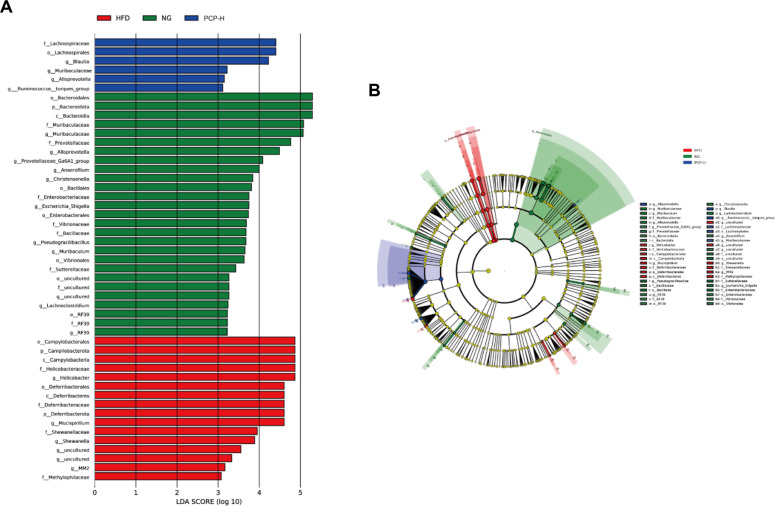
PCP affects gut microbiota profile. NG (control group), HFD (high-fat diet group), PCP-H (high-dose PCP group). (**A**) Histogram of LDA scores computed for feature differentially abundant. (**B**) Cladogram of LEfSe.

LEfSe and LDA were employed to analyze the difference in intestinal flora abundance across different groups. (Fig. 4A and 4B). A total of 27 genera predominantly belonging to the phylum *Bacteroidetes* were enriched in the control group, including *Bacteroidales*, *Muribaculaceae* and *Prevotellaceae*. On the other hand, the HFD group was enriched in 16 genera, including pathogenic bacteria such as *Helicobacteraceae* and *Deferribacterales*, whereas the PCP-H group was enriched in 6 genera primarily consisting of beneficial bacteria such as, *Lachnospirales*, *Muribaculaceae*, and *Blautia*. Collectively, these data indicate that PCP-H intervention promotes the growth of beneficial bacteria and suppresses the expansion of pathogenic bacteria in obese mice.

**Fig. 5:**
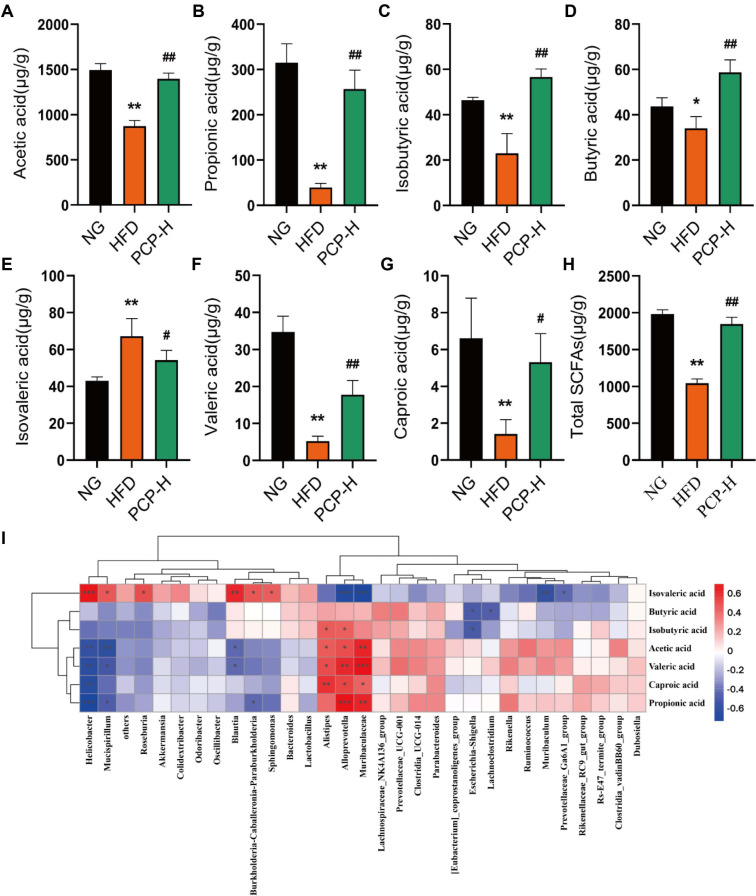
PCP affects SCFAs levels by regulating gut microbiota. NG (control group), HFD (high-fat diet group), PCP-H (high-dose PCP group). (**A**) acetic acid. (**B**) propionic acid. (**C**) isobutyric acid. (**D**) butyric acid. (**E**) isovaleric acid. (**F**) valeric acid. (**G**) caproic acid. (**H**) total SCFAs. (**I**) Correlation analysis between intestinal flora and SCFAs. Data were shown as mean ± SD (n =8). *p < 0.05, **p < 0.01 compared with Control group, and ^#^*p* < 0.05, ^##^*p* < 0.01 compared with HFD group.

SCFAs are products of microbial fermentation and play a crucial role in preventing and treating metabolic disorders. Compared with the control group, the HFD group had significantly decreased acetic acid, propionic acid, isobutyric acid, butyric acid, and caproic acid but increased isovaleric acid contents in the colon (all *p* < 0.05) (Fig. 5A-5H). However, these alterations were reversed following PCP-H intervention. We performed Spearman correlation analysis to evaluate the relationship between the changes in gut microbiota and SCFAs levels. Our data indicated that pathogenic bacteria such as *Helicobacter* and *Mucispirillum* were negatively correlated with acetic acid, propionic acid, and butyric acid levels, and positively correlated with isovaleric acid level. Conversely, beneficial bacteria including *Muribaculaceae*, *Alloprevotella* and *Lachnospiraceae_NK4A136*_group were positively correlated with acetic acid, propionic acid and butyric acid levels (Fig. 5I).

### PCP-H Alleviates Intestinal Inflammation and Enhances Intestinal Barrier Function

**Fig. 6:**
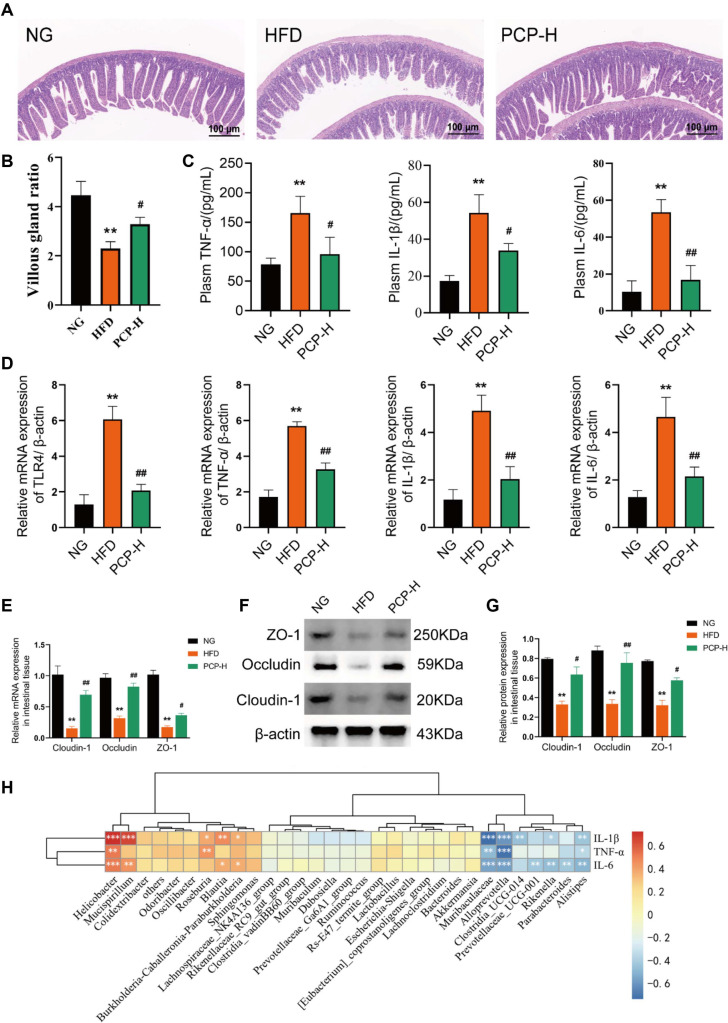
PCP alleviates intestinal inflammation and preserves intestinal barrier function in HFD-induced obese mice. NG (control group), HFD (high-fat diet group), PCP-H (high-dose PCP group). (**A**) Histomorphology of the colon. (**B**) Villous gland ratio. (**C**) Serum inflammatory factors TNF-α, IL-1β, and IL-6 (n =8). (**D**) Relative mRNA expressions of TLR4, TNF-α, IL-1β, and IL-6. (**E**) Relative mRNA expression in intestinal tissue. (**F**) Western blot analysis of ZO-1, occludin and claudin-1. (**G**) Relative protein expression in intestinal tissue (n =3). (**H**) Correlation analysis between genus level and inflammatory factors. *p < 0.05, **p < 0.01 compared with Control group, and ^#^*p* < 0.05, ^##^*p* < 0.01 compared with HFD group.

The effects of PCP-H on intestinal inflammation were evaluated by histopathology and the levels of proinflammatory cytokines. Compared with control mice, HFD-fed mice had disordered and sloughed villi with large intervillous spaces, shortened villi, increased crypt depth, and significantly decreased villus to crypt ratio (*p* < 0.01). In contrast, PCP-H administration in HFD-fed mice markedly increased villi length, decreased crypt depth, and increased villus to crypt ratio (Fig. 6A and 6B). Furthermore, the mRNA expression levels of inflammatory factor, including TNF-α, IL-1β, IL-6 and TLR4 in the intestinal tract were significantly higher in the HFD group compared to the control group, and significantly lower in the PCP-H group compared to the HFD group (Fig. 6D). Similar trends were observed in the levels of inflammatory factors in the serum (*p* < 0.01) (Fig. 6C). To determine the effect of PCP-H on intestinal barrier functions, the intestinal expression levels of claudin-1, occludin, and ZO-1 were quantified by RT-PCR and Western blot. It was found that HFD significantly downregulated the mRNA and protein expression of claudin-1, occludin and ZO-1 compared to normal diet (*p* < 0.01) (Fig. 6E and 6G), suggesting increased intestinal permeability and compromised barrier integrity. However, PCP-H treatment significantly upregulated the expression of these genes and proteins (*p* < 0.05). Spearman correlation analysis was conducted to analyse the relationship between changes in intestinal flora of TOP 30 and inflammatory factor level. The results showed that *Helicobacter* and *Mucispirillum* in HFD group were significantly positively correlated with the inflammatory factors IL-1β and IL-6, while *Muribaculaceae* and *Alloprevotella* in PCP-H group were significantly negatively correlated with the inflammatory factors TNF-α, IL-1β and IL-6 (Fig. 6H). Altogether, these findings indicate that PCP-H has the potential to mitigate intestinal inflammation and enhance intestinal barrier function in HFD-induced obese mice.

## Discussion

Our previous work has demonstrated that PCP is effective for preventing obesity. In this study, we further observed that PCP-H intervention effectively mitigated weight gain and decreased blood lipid, and blood glucose in obese mice. Furthermore, we showed that the protective effect of PCP-H in obesity is mediated through modulation of the gut microbiota.

The intestine is a complex microecological environment regulated by the homeostatic interplay among the intestinal barrier, microbiota, and contents. Gut microbes are a vital part of the intestinal microenvironment, and disturbances in the intestinal microbial ecosystem are closely associated with various metabolic disorders including obesity [26, 27]. Studies have shown that obese individuals have less rich and diverse gut microbiota than healthy individuals due to dysregulated lipid metabolism and increased body fat percentage [28]. Consistent with this, our findings confirmed that the microbiota of HFD-fed mice has decreased α- and β-diversity. In addition, PCP-H treatment increased the abundance and diversity of microbiota in obese mice, preliminarily proving that the impact of PCP-H on weight loss and lipid reduction may be linked to the restoration of gut dysbiosis.

The richness and diversity of the microbiota influence its composition and play an important role in the etiology and development of obesity. *Bacteroidetes* and *Firmicutes* are the predominant phyla in a healthy gut [29]. *Bacteroidetes* are considered beneficial probiotics, studies have shown that the relative abundance of *Bacteroidetes* in the gut of obese patients is generally low, and F/B is used as a biomarker for intestinal microbiome disturbances in obese patients [30, 31]. Moreover, *Proteobacteria* has been shown to exhibit pathogenic potentials and significantly impact host health [32], whereas *Desulfobacterota* may be associated with obesity [33]. Our study showed that PCP-H treatment significantly increased the relative abundance of *Bacteroides*, decreased F/B ratio, and reduced the relative abundance of *Proteobacteria* and *Desulfobacterota* in obese mice. In line with our results, Wei *et al*. demonstrated that HFD led to significantly increased relative abundance of *Desulfobacterota* and *Proteobacteria* in mice, which was reversed by treatment with polysaccharides from *Enteromorpha clathrata* [34]. *Muribaculaceae* is a family within the *Bacteroidetes* phylum consisting of beneficial bacterial species that significantly contribute to nutrient absorption and regulation of glucose and lipid metabolism [35]. *Alloprevotella*, *Prevotellaceae_UCG-001* and *Lachnospiraceae_NK4A136* are able to break down indigestible foods and generate energy for the host [36, 37]. Analysis of microbiota composition revealed that PCP-H treatment reversed the decreases in *Muribaculaceae*, *Alloprevotella*, *Lachnospiraceae_NK4A136*, and *Prevotellaceae_UCG-001*, as well as the increases in *Mucispirillum*, *Helicobacter* and *Escherichia*-*Shigella* induced by HFD. In addition, LEfSe and LDA analyses revealed that *Escherichia*-*Shigella* was enriched in the NG group, suggesting that the abundance of this genus may be regulated by diet type. The complex carbohydrates (e.g., cereal fiber) abundant in the normal diet may provide favorable conditions for its colonization[38]. The low abundance of *Escherichia*-*Shigella* in the PCP-H group may be attributed to the higher level of SCFAs produced by PCP-H via microbial fermentation, which reduce intestinal pH and thereby indirectly inhibit its proliferation[39]. Nevertheless, the specific mechanisms by which PCP-H regulates *Escherichia*-*Shigella* require further investigation.

SCFAs are major metabolites produced from the fermentation of carbohydrates by the gut microbiota, contributing significantly to the regulation of normal physiological processes within the intestinal tract [40, 41]. The primary components of SCFAs include acetic acid, propionic acid, and butyric acid [42]. Acetic acid has been shown to enhance glucose tolerance and insulin secretion in mice fed an HFD, while higher concentrations of acetic acid have been found to suppress lipid synthesis [43, 44]. Propionic acid can improve pancreatic function and is used by the liver for cholesterol metabolism [45]. In the present study, PCP-H treatment resulted in elevated levels of acetic acid, propionic acid, and butyric acid compared to HFD alone. In addition, *Helicobacter* has been reported to be inhibited by propionic acid [46], and *Lachnospiraceae* plays a crucial role in the production of butyric acid [47]. Research has indicated that *Alloprevotella* has the capacity to generate acetic acid, which attenuates inflammation and strengthens the intestinal barrier [48].The results of Spearman correlation analysis indicated a positive correlation between *Alloprevotella* and acetic acid, a negative correlation between *Helicobacter* and propionic acid, and a positive correlation between *Lachnospiraceae_NK4A136* and butyric acid. Thus, the increases in acetic acid, propionic acid and butyric acid production following PCP-H groups may be attributed to the abundance of *Alloprevotella*, *Helicobacter* and *Lachnospiraceae_NK4A136*. Together, these findings suggest that PCP-H is fermented and utilized by the gut microbiota to generate SCFAs, which help protect the host from obesity.

*Mucispirillum* are pathogenic bacteria that trigger intestinal inflammation [49]. *Helicobacter* and *Escherichia* (such as *E. coli*) are representative Gram-negative pathogens of the phylum *Proteobacteria*. Studies have shown that *Helicobacter* and *Escherichia* infections can lead to decreased immunity and trigger inflammation in the host due to the presence of lipopolysaccharides (LPS) on their outer membrane [32, 50, 51] Binding of LPS from Gram-negative bacterial to Toll-like receptor 4 (TLR-4) stimulates the secretion of various proinflammatory cytokines, causing intestinal inflammation [52, 53]. This process in turn alters the expression of tight junctions between IECs and increases intestinal permeability [54, 55]. Consequently, the compromised intestinal barrier allows pathogenic bacteria to enter the systemic circulation, leading to chronic low-grade inflammation. In line with this, we observed that obese mice had significantly increased abundance of Gram-negative bacteria (*e.g.*, *Proteobacteria*, *Helicobacter* and *Escherichia*-*Shigella*), upregulated intestinal mRNA expression of TLR-4, TNF-α, IL-1β, and IL-6. Serum levels of these inflammatory factors showed the same trend. In addition, the HFD-induced decrease in the gene and protein expression of c claudin-1, occludin and ZO-1 in the colon were markedly counteracted by PCP treatment. Therefore, it can be speculated that PCP-H reduced the relative abundance of pathogenic bacteria such as *Helicobacter* and *Mucispirillum*, while increasing the relative abundance of beneficial bacteria, including *Muribaculaceae* and *Alloprevotella*. This modulation of the gut microbiota may inhibit the release of intestinal pro-inflammatory factors, alleviate local intestinal inflammation repair intestinal barrier function and reduce serum inflammatory level.

Taken together, these findings suggest that PCP-H exerts its protective effects through a multi-step axis involving modulation of gut microbiota, enhancement of intestinal barrier function, and suppression of local and systemic inflammation. This axis - “polysaccharide - gut microbiota - SCFAs - intestinal barrier/inflammation” - is highly consistent with key pathological processes driving insulin resistance and obesity in humans[56], suggesting cross-species relevance. Furthermore, although the composition of the gut microbiota may vary across species, the metabolic benefits of PCP as an oral dietary agent via restoring microbial homeostasis provide clear preclinical support for its development as a prebiotic or functional food against human obesity. Further validation of its efficacy and mechanisms could be pursued through microbiota transplantation, alternative animal models, and preliminary clinical studies.

Nevertheless, despite these promising findings, several limitations should be acknowledged in this study. The number of experimental animals was determined mainly based on previous literature and our preliminary experience, without strict a priori sample size calculation. Blinding was not applied during sample processing and data analysis, which may introduce potential bias. Although the high-fat diet-induced obese C57BL/6J mouse model is widely accepted and classic, it still cannot fully recapitulate the complex pathological characteristics of human obesity. These limitations will be further addressed and improved in future studies.

## Conclusion

Our study demonstrates that PCP-H alleviates obesity through the modulation of the gut microbiota. PCP-H restores HFD-induced gut dysbiosis and promotes the proliferation of beneficial bacteria, thereby increasing the production of SCFAs and preventing obesity. Additionally, PCP-H may restore intestinal barrier function by inhibiting the growth of pathogenic bacteria and inhibiting the release of pro-inflammatory cytokines. These findings indicate that PCP is a promising candidate for the treatment of obesity.

## Supplemental Materials

Supplementary data for this paper are available on-line only at http://jmb.or.kr.



## Figures and Tables

**Table 1:** Composition of high-fat-diet.

Composition	Weight (g)	Energy (Kcal)
Tyrosine	200	800
L-cystine	3	12
Maltodextrin 10	125	500
Saccharose	68.8	275.2
Cellulose	50	0
Soybean oil	25	225
Lard	245	2205
Mineral mixture S100026	10	0
Calcium hydrogen Phosphate	13	0
Calcium carbonate	5.5	0
Potassium citrate	16.5	0
Vitamin mixture	10	40
Choline bitartrate	2	0
Total	773.85	4075
