# Supplementary material for: Polygonatum cyrtonema Polysaccharides Alleviates Diet-Induced Obesity in Mice by Modulating the Gut Microbiota and Reducing Intestinal Inflammation
Source: J Microbiol Biotechnol. 2026 May 15;36:e2512028. doi: 10.4014/jmb.2512.12028 (PMC13184603; doi:10.4014/jmb.2512.12028)
Supplement: Supplementary file 1 [file jmb-36-e2512028-supple.pdf]

J. Microbiol. Biotechnol. 2026. 36: e2512028
https://doi.org/10.4014/jmb.2512.12028

Supplementary Tables

Tabld S1. Primers used for quantitative RT- PCR analysis of intestinal samples.

Gene Name	Primer	Primer Sequence (5'→3')
Claudin-1	Forward primer	GGCTTCTCTGGGATGGATCG
	Reverse primer	TTTGCGAAACGCAGGACATC
Occludin	Forward primer	CCCCTCTTTCCTTAGGCGAC
	Reverse primer	TTCAAAAGGCCTCACGGACA
ZO-1	Forward primer	GAGCAGGCTTTGGAGGAGAC
	Reverse primer	TGGGACAAAAGTCCGGGAAG
IL-1β	Forward primer	GAAATGCCACCTTTTGACAGTG
	Reverse primer	TGGATGCTCTCATCAGGACAG
IL-6	Forward primer	GGAGCCCACCAAGAACGATA
	Reverse primer	ACCAGCATCAGTCCCAAGAA
TNF-α	Forward primer	CTCATGCACCACCATCAAGG
	Reverse primer	ACCTGACCACTCTCCCTTTG
TLR4	Forward primer	AAGTGGCCCTACCAAGTCTC
	Reverse primer	CTGCAGCTCTTCTAGACCCA
β-actin	Forward primer	AGTGTGACGTTGACATCCGT
	Reverse primer	TGCTAGGAGCCAGAGCAGTA

Table S2. Primers used for quantitative RT- PCR analysis of liver and epididymal fat.

Gene Name	Primer	Primer Sequence (5'→3')
DGAT1	Forward primer	TGGTAGTGGGCCCAAGGTAG
	Reverse primer	TGCAGACGATGGCACCTCAG
FAS	Forward primer	GTCAACCATGCCAACCTGAAAA
	Reverse primer	AATCACTCCAACGGGCTGAA
ACC	Forward primer	GAGGCGGATATCTGCTGAGAC
	Reverse primer	GGAGTGCTGGTTTAGCTCCA
CYP51	Forward primer	CACACATTGCCACAGGGAGA
	Reverse primer	GAAGTGGCCCAACTACACGA
PPARα	Forward primer	TGGTGTTCGCAGCTGTTTTG
	Reverse primer	AGATACGCCCAAATGCACCA
CPT1α	Forward primer	GGACTCCGCTCGCTCATTC
	Reverse primer	AGGCAGATCTGTTTGAGGGC
HMGCR	Forward primer	AGAGAACAAGGGTTCACGCC
	Reverse primer	CCTTGGATCCCACGCGGA
DGAT2	Forward primer	ACTGGAACACGCCCAAGAAA
	Reverse primer	GTAGTCTCGGAAGTAGCGCC
LPL	Forward primer	GCCTTTCTCCTGATGACGCT
	Reverse primer	GCAATCACACGGATGGCTTC
SREBP-1α	Forward primer	GGGGCCTGACAGGTGAAATC
	Reverse primer	TGAGCTGGAGCATGTCTTCAAA
β-actin	Forward primer	AGTGTGACGTTGACATCCGT
	Reverse primer	TGCTAGGAGCCAGAGCAGTA

Table S3. Normal chow composition.

Ingredient	Weight (g)	Energy (Kcal)
Corn	380	850.20
Soybean meal	200	153.69
Wheat bran	120	268.48
Wheat middlings	190	425.10
Fish meal	22	16.91
Soybean oil	12	4.97
Dicalcium phosphate	20	0.00
Choline chloride	4	0.00
L-Lysine	11.1	8.53
DL-Methionine	4.5	3.46
L-Cystine	6.4	4.92
Mineral mixture	10	0.00
Vitamin mixture	10	0.00
Total	990	1736.26
